# Impact of Fusion Partners and Transplantation Benefit in Intensively Treated *KMT2A*-Rearranged Acute Myeloid Leukemia

**DOI:** 10.3390/cancers18030401

**Published:** 2026-01-27

**Authors:** Heng Shen, Jiayuan Chen, Xiaoyuan Gong, Chunlin Zhou, Dong Lin, Kaiqi Liu, Benfa Gong, Guangji Zhang, Yan Li, Yuntao Liu, Shaowei Qiu, Bingcheng Liu, Ying Wang, Yingchang Mi, Qiuyun Fang, Jianxiang Wang, Hui Wei

**Affiliations:** 1 State Key Laboratory of Experimental Hematology, National Clinical Research Center for Blood Diseases, Haihe Laboratory of Cell Ecosystem, Institute of Hematology & Blood Diseases Hospital, Chinese Academy of Medical Sciences & Peking Union Medical College, Tianjin 300020, China; shenheng@ihcams.ac.cn (H.S.); chenjiayuan@ihcams.ac.cn (J.C.); gongxiaoyuan@ihcams.ac.cn (X.G.); zhouchunlin@ihcams.ac.cn (C.Z.); lindong@ihcams.ac.cn (D.L.); liukaiqi@ihcams.ac.cn (K.L.); gongbenfa@ihcams.ac.cn (B.G.); zhangguangji@ihcams.ac.cn (G.Z.); liyan1@ihcams.ac.cn (Y.L.); liuyuntao@ihcams.ac.cn (Y.L.); qiushaowei@ihcams.ac.cn (S.Q.); liubingcheng@ihcams.ac.cn (B.L.); wangying1@ihcams.ac.cn (Y.W.); ychmi@ihcams.ac.cn (Y.M.); fangqiuyun@ihcams.ac.cn (Q.F.); 2 Tianjin Institutes of Health Science, Tianjin 301600, China

**Keywords:** acute myeloid leukemia, *KMT2A* rearrangement, hematopoietic stem cell transplantation, clinical outcomes

## Abstract

Background: *KMT2A* rearrangements are a frequent genetic abnormality associated with Acute myeloid leukemia (AML), historically linked to varied prognoses and outcomes. The prognosis for patients with this rearrangement remains controversial, necessitating further research to stratify risk and guide treatment. Methods: In this retrospective study, a total of 3468 adolescent and adult AML patients were screened, and 181 patients harboring *KMT2A* rearrangements were analyzed. We used FISH, RT-PCR, and next-generation sequencing, including transcriptome and targeted panels, for diagnosis and mutation profiling. All patients received intensive chemotherapy. We evaluated overall survival and event-free survival using Kaplan–Meier and Cox regression models, with HSCT analyzed as a time-dependent variable. Results: The incidence of *KMT2A*-rearranged AML in our newly diagnosed cohort was 5.9%. Among the 181 patients included in the final analysis, 89 (49.2%) were male and 92 (50.8%) were female, with a median age of 33 years (range: 13–65). The distribution of fusion partners included *KMT2A*::*MLLT3* (*n* = 39), *KMT2A*::*AFDN* (*n* = 27), *KMT2A*::*MLLT10* (*n* = 25), *KMT2A*::*ELL* (*n* = 24), and others (*n* = 12). Seventy-four patients underwent allogeneic hematopoietic stem cell transplantation (allo-HSCT) in first complete remission (CR1). The median follow-up for survivors was 17.53 months (range 1.47–112.57), and the 3-year overall survival (OS) and event-free survival (EFS) for the entire cohort were 42.0% and 32.1%, respectively. Patients with *KMT2A*::*ELL* exhibited superior OS compared to other subtypes (3-year OS [ELL vs. non-ELL]: 59.8% vs. 39.3%, *p* = 0.023). Concomitant mutations did not significantly impact the prognosis of *KMT2A*-rearranged AML patients. In multivariate analysis, age and HSCT in CR1 were independently associated with OS and EFS (OS: HR = 1.022, *p* = 0.026 [age]; HR = 0.238, *p* < 0.001 [HSCT]; EFS: HR = 1.027, *p* = 0.002 [age]; HR = 0.155, *p* < 0.001 [HSCT]). Patients aged over 20 years were more likely to benefit from HSCT than those aged 20 years or younger (*p* < 0.001 [age > 20], *p* = 0.780 [age ≤ 20]). Conclusions: Our study revealed the heterogeneous outcomes of *KMT2A*-rearranged AML patients and clarified the impact of HSCT across different age groups.

## Simple Summary

Acute myeloid leukemia with *KMT2A* rearrangements is challenging, characterized by diverse clinical outcomes. Given the varied clinical outcomes across different *KMT2A* fusion subtypes, the specific benefit of hematopoietic stem cell transplantation (HSCT) for each subgroup remains under-investigated, which is critical for making precise treatment decisions in the era of emerging targeted therapies. In this study, we analyzed 181 *KMT2A*-rearranged patients to determine how different fusion partners affect clinical outcomes. We found that patients with *KMT2A*::*ELL* had better outcomes compared to others. Crucially, our research revealed that the benefit of HSCT is highly dependent on patient age. While HSCT significantly improved survival for patients over the age of 20, it did not provide a statistically significant survival advantage for patients aged 20 or younger. We suggest that treatment decisions, especially regarding HSCT, should be personalized based on both the specific genetic characteristics and the age of the patient to optimize survival chances.

## 1. Background

Acute myeloid leukemia (AML), the most prevalent acute leukemia in adults, is stratified into different risk groups based on the 2022 European Leukemia Net (ELN) and National Comprehensive Cancer Network (NCCN) guidelines [1,2,3]. The *KMT2A* (lysine methyltransferase 2A) gene, also known as the *MLL* (mixed lineage leukemia) gene, is located in the 11q23.3 region of human chromosomes. *KMT2A* rearrangements are a reproducible genetic abnormality in acute myeloid leukemia (AML) [3,4,5,6]. To date, more than 130 different recombinations in *MLL* have been identified. *AFF1*, *MLLT3*, *MLLT1*, *MLLT10*, *AFDN*, and *ELL* were the most common fusion partners identified in *KMT2A*-rearranged AML, observed in over 90% of cases [7,8,9,10]. According to the ELN 2022 and NCCN guidelines, AML with *KMT2A*::*MLLT3* is classified as intermediate-risk, while those with other *KMT2A*-rearranged subtypes are classified as adverse-risk [3,8].

While recent studies largely support the prognostic stratification proposed by the ELN 2022 guidelines, clinical outcomes for specific fusion partners remain heterogeneous, and the benefit of allogeneic HSCT across these diverse subgroups requires further investigation [11,12,13]. Previous studies have demonstrated that adult patients with *KMT2A*-rearranged AML could benefit from allogeneic hematopoietic stem cell transplantation (allo-HSCT) [14,15,16,17]. However, few studies have explored the potential benefit of HSCT for each *KMT2A*-rearrangement partner subgroup. Particularly in the era of emerging targeted therapies such as menin inhibitors, it is vital to understand the baseline benefit of HSCT in different subtypes to make precise therapeutic decisions.

Consequently, our study sought to investigate outcomes and potential factors influencing prognosis in AML patients with *KMT2A* rearrangements. Additionally, we conducted a subgroup analysis to identify populations of *KMT2A*-rearranged AML patients who might benefit from allo-HSCT for improved survival.

## 2. Methods

### 2.1. Patient Selection

We retrospectively assessed 3468 consecutive adolescent and adult patients with newly diagnosed AML treated at the Institute of Hematology and Blood Diseases Hospital of the Chinese Academy of Medical Sciences (Tianjin, China) between October 2010 and April 2024. The enrolled patients had to meet the following criteria: (1) diagnosed as *KMT2A*-rearranged AML through karyotype, Fluorescence In Situ Hybridization (FISH), Reverse Transcription Polymerase Chain Reaction (RT-PCR), or transcriptome sequencing; (2) receiving intensive chemotherapy. This study was approved by the Ethics Committee of the Institute of Hematology and Blood Diseases Hospital of the Chinese Academy of Medical Sciences (Tianjin, China) and conducted in accordance with the Declaration of Helsinki.

### 2.2. FISH, RT-PCR and NGS

Fluorescence in situ hybridization (FISH) was performed on bone marrow mononuclear cells by our clinical center laboratory. Split-signal assays were conducted using the Vysis LSI MLL Dual Color, Break Apart Rearrangement Probe (Abbott Molecular, Des Plaines, IL, USA) to detect 11q23/KMT2A gene rearrangements. The assay was performed according to the manufacturer’s instructions.

For RT-PCR, total RNA was extracted from bone marrow. Supplementary Table S1 lists the panel of fusion genes detected by RT-PCR, which were associated with hematologic malignancies. PCR amplification was performed as described previously [18,19].

All the targeted NGS data mentioned in this article was obtained from our clinical center. The targeted sequencing panel covered 267 common genes (Supplementary Table S2) in hematologic malignancies. The panel design was undertaken at the Clinical Testing Center of the Institute of Hematology and Blood Diseases Hospital (Tianjin, China), and the detailed protocol has been described previously [19,20]. The sequencing data was processed into vcf files with the reference genome hg19 at the Clinical Testing Center. ANNOVAR software (version: 8 Jun 2020. Center for Applied Genomics, Children’s Hospital of Philadelphia, Philadelphia, PA, USA) [21] was used for variant annotation. All mutation sites were filtered using Integrative Genomics Viewer (IGV, version: 2.15.1 Broad Institute, Cambridge, MA, USA) [22] to eliminate false positives.

### 2.3. Treatments

Enrolled patients received intensive induction therapy with one of the following regimens: (1) DA: Daunorubicin (60 mg/m^2^/day, IV, days 1–3) and cytarabine (100 mg/m^2^/day, IV, days 1–7); (2) HAD: Homoharringtonine (2 mg/m^2^/day, IV, days 1–7), daunorubicin (40 mg/m^2^/day, IV, days 1–3), and cytarabine (100 mg/m^2^/day, IV, days 1–7); and (3) DAV: Venetoclax was initiated with a 2-day ramp-up phase (100 mg and 200 mg) prior to chemotherapy, followed by 400 mg daily on days 1–7, in combination with daunorubicin (60 mg/m^2^/day, IV, days 1–3) and cytarabine (100 mg/m^2^/day, IV, days 1–7). As detailed in our previous studies [19,23], the therapeutic protocols were consistent with the standard of care at our center. Post-remission management was stratified based on transplant eligibility. For transplant-eligible patients, donor searching was initiated immediately. During the interval, patients received one or two courses of intermediate- or high-dose cytarabine as bridging therapy to sustain remission. HSCT was performed as soon as a donor became available. Patients precluded from HSCT due to donor unavailability or patient preference received standard consolidation chemotherapy for a total of 3 courses. Routine prophylaxis against central nervous system (CNS) leukemia was administered via intrathecal injection. The regimen consisted of methotrexate (10 mg), cytarabine (50 mg), and dexamethasone (10 mg) per dose. In general, two doses were administered during induction and each consolidation course, as described in our previous studies [18,19]. Supportive care regarding infection management was described in our previous studies [23]. Antifungal prophylaxis, such as posaconazole, voriconazole, or caspofungin, was administered based on the attending physician’s clinical experience and judgment. Routine antibacterial prophylaxis was not employed; instead, antibiotic treatment was administered upon the onset of symptoms, based on pathogenic microbiological examination and drug susceptibility results.

### 2.4. Statistical Analysis

Descriptive statistics were employed to outline patients’ baseline characteristics. Differences in baseline characteristics were assessed using the Chi-squared test or two-sided Fisher’s exact test for categorical variables and the Mann–Whitney U test for continuous variables. OS was defined as the interval from the date of diagnosis to death from any cause or the last follow-up. EFS was defined as the time from diagnosis to induction failure, relapse, death in CR, or last follow-up, whichever occurred first. The optimal cutoff values for continuous variables, including age and WBC count, were determined using the “surv_cutpoint” function in R. Landmark analysis was utilized to prevent bias introduced by patients who experienced early relapse or death before HSCT when analyzing the effect of HSCT. The landmark day was set as the median time from the date of first CR to HSCT. OS and EFS were evaluated by the Cox or Kaplan–Meier method and compared with the Log-rank test. HSCT was treated as a time-dependent variate in the Cox regression model. A *p*-value of less than 0.05 was deemed statistically significant. All statistical analyses were conducted using IBM SPSS version 26 (IBM Corp, Armonk, NY, USA) and R version 4.2.0 (R Foundation for Statistical Computing, Vienna, Austria).

## 3. Results

### 3.1. Patient Characteristics

**Figure 1:**
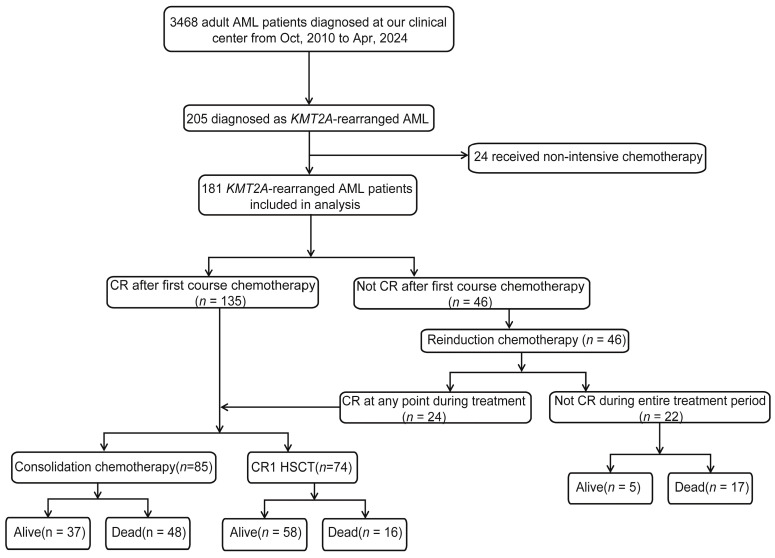
Flowchart of the KMT2A-rearranged AML cohort and treatment outcomes.

The incidence of *KMT2A*-rearranged AML in our cohort with newly diagnosed AML was 5.9% (205/3468). Of the 205 identified *KMT2A*-rearranged patients, 24 patients were excluded from the final analysis as they received non-intensive chemotherapy including hypomethylating agents or palliative care. Consequently, a total of 181 patients who underwent intensive induction chemotherapy between 1 October 2010 and 1 April 2024 were included in this study. The screening process and criteria are shown in Figure 1. Among these 181 patients, 89 (49.2%) were male and 92 (50.8%) were female, with a median age of 33 (range 13–65) years. According to the partner genes of *KMT2A* fusions, 24 (13.3%) patients were *ELL*, 27 (14.9%) were *AFDN*, 39 (21.5%) were *MLLT3*, 25 (13.8%) were *MLLT10*, and 12 (6.6%) had other rare types of *KMT2A* rearrangement. The type of *KMT2A* rearrangements could not be determined in 54 (29.8%) patients, because their results were only available for FISH detection. The detailed distribution of these fusion partners is illustrated in Supplementary Figure S1. Genetic testing information via targeted NGS was evaluable in 128 patients from our cohort. The most common concomitant mutations included *K-RAS* (*n* = 41, 32.0%), *N-RAS* (*n* = 40, 31.3%), *PTPN11* (*n* = 20, 15.6%) and *FLT3* (*n* = 19, 14.8%). A total of 74 (40.9%) patients underwent HSCT following the first complete remission (CR1). Detailed clinical characteristics are shown in Table 1.

To assess whether baseline clinical characteristics were balanced among different *KMT2A*-rearrangement subtypes, we summarized in Supplementary Table S3. The ‘Unknown’ group (*n* = 54) was excluded due to missing mutation data for 39 patients. Age and gender distributions were balanced across the *KMT2A*::*MLLT3*, *KMT2A*::*AFDN*, *KMT2A*::*MLLT10*, *KMT2A*::*ELL*, and “other” groups. However, the initial WBC count in the *KMT2A*::*AFDN* group was significantly higher than other groups (*p* < 0.001). Notably, there was no significant difference in the distribution of intensive induction chemotherapy regimens among the subtypes (*p* = 0.330).

**Figure 2:**
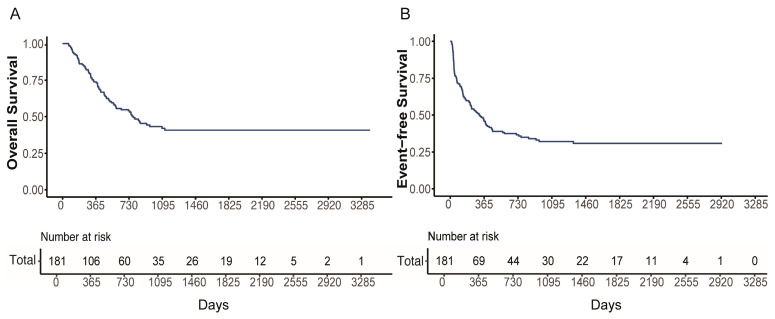
Survival analyses of KMT2A-rearranged AML cohort. (**A**) OS and (**B**) EFS of 181 patients with KMT2A-rearranged AML.

Of 181 patients, the median follow-up for survivors was 17.53 months (1.47–112.57), and the 3-year OS and EFS of the entire cohort were 42.0% (95% CI, 34.1–51.8%) and 32.1% (95% CI, 25.3–40.6%), respectively (Figure 2A,B).

### 3.2. Impact of KMT2A Rearrangement Subtypes on Outcomes

**Figure 3:**
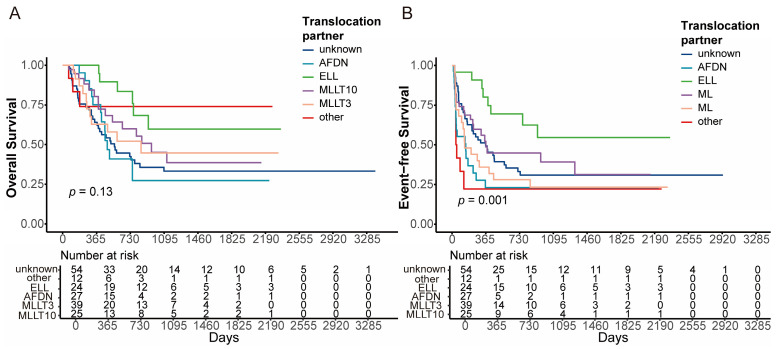
Survival analyses stratified by specific *KMT2A* fusion partners. Kaplan–Meier curves for (**A**) OS and (**B**) EFS. The *p*-values indicate the global significance across all subgroups, calculated using the Log-rank test.

According to the partner genes of *KMT2A* rearrangement, the 3-year OS was 59.8% (95% CI, 38.7–92.3%), 27.3% (95% CI, 10.3–72.3%), 45.1% (95% CI, 28.7–71.0%), and 44.7% (95% CI, 26.7–74.9%) in the *ELL* (*n* = 24), *AFDN* (*n* = 27), *MLLT3* (*n* = 39), and *MLLT10* (*n* = 25) groups, respectively (global *p* = 0.130, Figure 3A). The 3-year EFS was 54.7% (95% CI, 35.0–85.4%), 23.0% (95% CI, 10.9–48.8%), 39.2% (95% CI, 24.6–62.7%) and 23.3% (95% CI, 11.3–48.1%) in the *ELL*, *AFDN*, *MLLT3*, and *MLLT10* groups, respectively (global *p* = 0.001, Figure 3B). *KMT2A*::*ELL* was associated with better OS and EFS compared to those with other partner genes (OS, *p* = 0.023; EFS, *p* = 0.003, Supplementary Figure S2A,B). No significant differences in either OS or EFS were observed in the *MLLT3* subgroup compared to the other groups (OS, *p* = 0.640; EFS, *p* = 0.430, Supplementary Figure S2C,D).

After setting HSCT as a censoring event, the 3-year OS and EFS for the entire cohort were 21.9% (95% CI, 13.5–35.3%) and 15.6% (95% CI, 8.7–28.2%), respectively (Supplementary Figure S3A,B). The 3-year OS was 30.0% (95% CI, 6.3–100.0%), 13.3% (95% CI, 2.5–71.4%), 18.9% (95% CI, 5.6–64.7%), and 11.9% (95% CI, 1.9–73.8%) in the *ELL* (*n* = 24), *AFDN* (*n* = 27), *MLLT3* (*n* = 39), and *MLLT10* (*n* = 25) groups, respectively (*p* = 0.27, Supplementary Figure S4A). The 3-year EFS was 25.7% (95% CI, 5.3–100.0%), not reach (NR), 19.8% (95% CI, 6.2–62.8%) and NR in the *ELL*, *AFDN*, *MLLT3*, and *MLLT10* groups, respectively (*p* < 0.001, Supplementary Figure S4B). Patients with *KMT2A*::*ELL* demonstrated superior EFS and a trend towards OS compared with non-ELL subtypes (OS, *p* = 0.053; EFS, *p* = 0.001; Supplementary Figure S4C,D). *KMT2A*::*MLLT3* still did not exhibit significant differences in OS or EFS when compared to other groups (OS, *p* = 0.470; EFS, *p* = 0.560; Supplementary Figure S4E,F).

### 3.3. Prognostic Factors for KMT2A-Rearranged AML

To exclude the potential confounding effect of historical advances in supportive care, we stratified the patients into two cohorts based on year of diagnosis: the earlier era (2010–2016) and the recent era (2017–2024). Patients diagnosed in the recent era exhibited significantly superior OS compared to those in the earlier era (3-year OS: 33.2% [95%CI, 23.0–48.1%] vs. 48.1% [95%CI, 37.4–62.0%], *p* = 0.045, Supplementary Figure S5A). However, no significant difference was observed in EFS between the two eras (3-year EFS: 29.8% [95%CI, 20.0–44.4%] vs. 33.5% [95%CI, 24.8–45.3%], *p* = 1.000, Supplementary Figure S5B).

We further performed univariate Cox analysis to explore other prognostic factors, including year of diagnosis and co-mutations (Supplementary Table S4). Regarding the year of diagnosis, patients treated in the recent era (2017–2024) showed superior OS compared to the earlier era (HR = 0.641, 95% CI 0.414–0.994, *p* = 0.047), whereas no significant association was observed with EFS (HR = 0.999, 95% CI 0.687–1.474, *p* = 0.998). Notably, the five most frequently mutated genes, including *KRAS*, *NRAS*, *PTPN11*, *FLT3*, and *WT1*, were not significantly associated with OS or EFS. Age and initial WBC were significant prognostic factors for both OS and EFS (Age: OS, HR = 1.020, 95% CI 1.000–1.043, *p* = 0.045; EFS, HR = 1.021, 95% CI, 1.004–1.038, *p* = 0.013; WBC: OS, HR = 1.003, 95% CI 1.000–1.007, *p* = 0.050; EFS, HR = 1.005, 95% CI, 1.002–1.008, *p* = 0.001). Setting *KMT2A*::*ELL* as a reference, univariate analysis confirmed its favorable prognostic role. The *KMT2A*::*AFDN* subgroup exhibited the poorest outcomes, with a significantly increased risk for both OS (HR = 2.926, 95% CI 1.093–7.831, *p* = 0.033) and EFS (HR = 4.322, 95% CI 1.884–9.916, *p* = 0.001). The *KMT2A*::*MLLT10* subgroups also showed significantly inferior EFS (HR = 3.310, 95% CI 1.448–7.569, *p* = 0.005), whereas no significant difference was observed in OS (HR = 2.275, 95% CI 0.841–6.155, *p* = 0.106). While the outcome of *KMT2A*::*MLLT3* was comparable to *KMT2A*::*ELL* (OS, HR = 2.039, 95% CI 0.791–5.259, *p* = 0.141; EFS, HR = 2.188, 95% CI, 0.969–4.944, *p* = 0.060).

**Figure 4:**
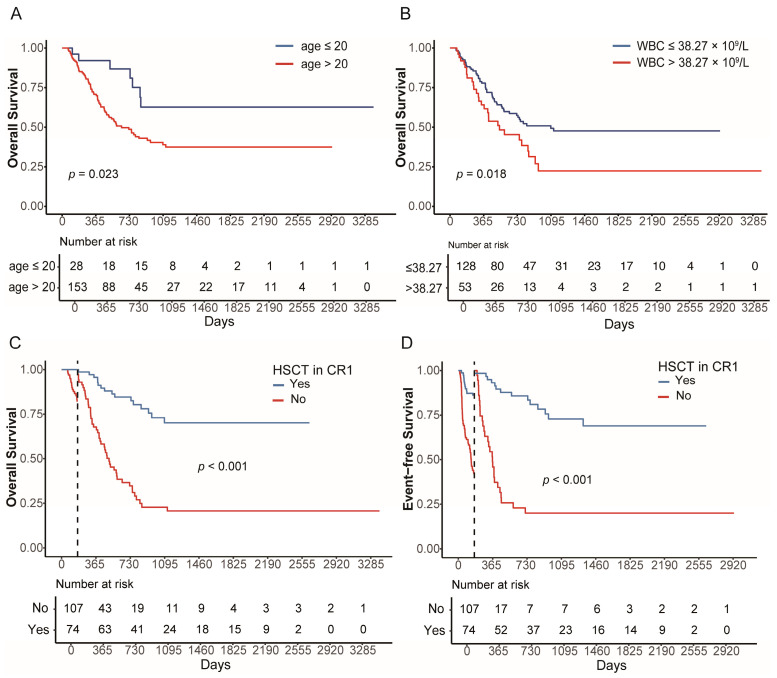
Survival analyses according to prognostic factors. (**A**) OS stratified by age (**A**) and WBC count at diagnosis (**B**). Landmark analysis of OS (**C**) and EFS (**D**) comparing the role of HSCT in KMT2A-rearranged AML. The vertical dashed line indicates the landmark time point, defined as the median time from first CR to HSCT.

We used the “surv_cutpoint” function in R to identify the optimal cutoff value for age and WBC. Consequently, we divided the cohort into two groups: age > 20 (*n* = 153) and age ≤ 20 (*n* = 28). Significant differences in OS were identified between the two groups, with 3-year OS of 38.9% (95% CI, 30.5–49.5%) and 62.6% (95% CI, 43.7–89.9%), respectively (Figure 4A). Similarly, we divided the cohort into two groups according to WBC: WBC > 38.27 × 10^9^/L (*n* = 53) and WBC ≤ 38.27 × 10^9^/L (*n* = 128). The 3-year OS of the two groups was 22.4% (95% CI, 11.3–44.4%) and 47.7% (95% CI, 38.4–59.3%), respectively (Figure 4B). Concomitant mutations did not have a significant impact on the prognosis of *KMT2A*-rearranged AML patients. No significant differences were observed in baseline clinical characteristics between transplanted and non-transplanted patients (Table 1). The time-dependent univariate Cox analysis indicated that HSCT in CR1 significantly improved OS and EFS (OS: HR = 0.221, 95% CI 0.125–0.391, *p* < 0.001; EFS: HR = 0.194, 95% CI, 0.101–0.371, *p* < 0.001). The landmark day was set as the median time from the date of first CR to HSCT. Landmark analysis also showed that HSCT significantly improved OS and EFS in *KMT2A*-rearranged AML patients (OS: *p* < 0.001; EFS: *p* < 0.001, Figure 4C,D).

Finally, we included age, WBC, *KMT2A*-rearrangement subtype, the year of diagnosis and HSCT in CR1 in the multivariate analysis. Notably, the multivariate analysis indicated that the year of diagnosis was not independently associated with either OS (HR = 0.660, 95% CI 0.392–1.192, *p* = 0.118) or EFS (HR = 1.036, 95% CI 0.659–1.629, *p* = 0.878). The results indicated that age and HSCT in CR1 were independently associated with OS and EFS (OS: HR = 1.022, 95% CI 1.002–1.042, *p* = 0.029 [age]; HR = 0.233, 95% CI 0.129–0.422, *p* < 0.001 [HSCT in CR1]; EFS: HR = 1.027, 95% CI 1.010–1.044, *p* = 0.002 [age]; HR = 0.155, 95% CI 0.078–0.308, *p* < 0.001 [HSCT in CR1], Supplementary Table S4).

### 3.4. The Impact of HSCT in Subgroups of KMT2A-Rearranged AML

**Figure 5:**
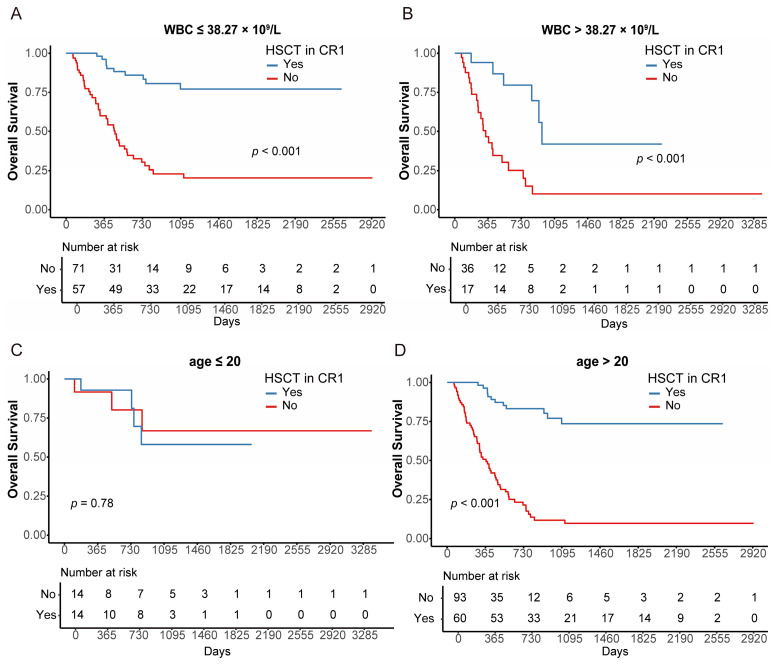
Survival analysis according to HSCT in CR1 in subgroups of KMT2A-rearranged AML patients. OS stratified by HSCT in CR1 of WBC ≤ 38.27 × 109/L (**A**), WBC > 38.27 × 109/L (**B**), age ≤ 20 (**C**) and age > 20 (**D**).

**Figure 6:**
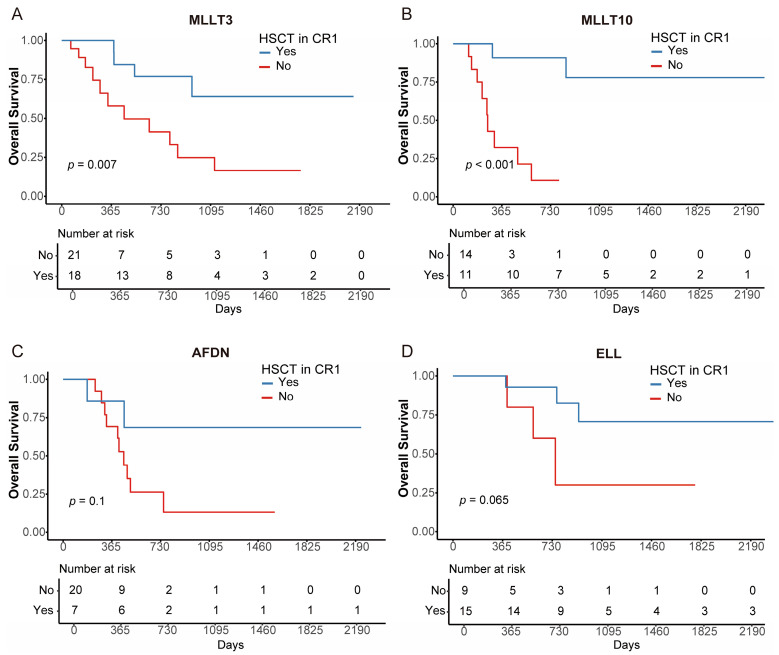
Survival analysis according to HSCT in CR1 in specific KMT2A-rearragned subtype. OS stratified by HSCT in CR1 of MLLT3 (**A**), MLLT10 (**B**), AFDN (**C**) and ELL (**D**).

To identify which patients would benefit from HSCT, we performed a subgroup analysis. We found that both patients with WBC > 38.27 × 10^9^/L and WBC ≤ 38.27 × 10^9^/L could significantly benefit from HSCT (both *p* < 0.001; Figure 5A,B). In addition, patients aged > 20 years were more likely to benefit from HSCT than those aged ≤ 20 years (*p* < 0.001 [age > 20], *p* = 0.780 [age ≤ 20]; Figure 5C,D). Regarding *KMT2A*-rearranged subtypes, significant advantages were observed in the *MLLT3* and *MLLT10* groups, with a trend in *ELL* and *AFDN* groups (HSCT vs. non-HSCT (3-year OS [95% CI]): 64.1% [40.2–100%] vs. 24.8% [9.4–65.6%], *p* = 0.007 [*MLLT3*]; 77.9% [54.6–100%] vs. 10.7% [1.7–67.7%], *p* < 0.001 [*MLLT10*]; 70.7% [47.1–100%] vs. 30.0% [6.31–100%], *p* = 0.065 [*ELL*]; 68.6% [40.3–100%] vs. 13.2% [2.45–71.1%], *p* = 0.1 [*AFDN*], Figure 6A–D).

## 4. Discussion

Our study presents a retrospective analysis of 181 patients with *KMT2A*-rearranged AML treated with intensive chemotherapy. We assessed the prognostic impact of heterogeneity within *KMT2A* rearrangements and investigated the survival benefit conferred by allo-HSCT. The outcomes of *KMT2A*-rearranged AML were dismal and were influenced by partner genes. HSCT in CR1 improved outcomes; however, the magnitude of benefit varied across patient age groups.

Our study underscores the significant influence of *KMT2A* rearrangement partner genes on survival outcomes in AML [24,25,26]. In line with prior literature, patients harboring *KMT2A*::*AFDN* and *KMT2A*::*MLLT10* fusions exhibited the poorest survival in our cohort [13,15,27], and we found better outcomes in the *KMT2A*::*ELL* compared with other *KMT2A*-rearranged subtypes. Conflicting results were reported for the outcomes of *KMT2A*::*ELL* AML. Chen et al. demonstrated that *KMT2A*::*ELL* patients exhibited significantly lower 5-year OS and EFS (both 6%) [28]. Tamai et al. demonstrated the unfavorable prognosis of t (11;19) patients, with 1-year EFS of 12.8% and 2-year OS of 10.7% [29]. Conversely, some studies indicate a more favorable prognosis for *KMT2A*::*ELL* AML. Grimwade et al. have reported that *KMT2A*::*ELL* and *KMT2A*::*MLLT3* patients have an OS comparable to those with normal karyotype AML [13]. Wu et al. reported the best OS for *KMT2A*::*ELL* than other subtypes [30]. Recently, Zhang et al. reported that the *KMT2A*::*ELL* subgroup exhibited a better OS (3-year OS, 65.3%), although there was no statistically significant difference compared to other *KMT2A*-rearranged subtypes [31].

The survival outcomes of *KMT2A*::*MLLT3* patients in our cohort were comparable to those of other *KMT2A*-rearranged AML patients, although several studies have demonstrated that *KMT2A*::*MLLT3* exhibited better survival than other subtypes. The survival outcomes of *KMT2A*::*MLLT3* patients in our cohort aligned with previous studies, showing 3-year OS ranging from 35% to 45% [13,24,25,26,28]. In early reports from the Cancer and Leukemia Group B (CALGB), patients with *KMT2A*::*MLLT3* displayed significantly superior EFS compared to those with other *KMT2A*-rearranged subtypes [12]. The most recent report from CALGB showed that patients under 60 years showed a significantly improved 3-year OS in the *KMT2A*::*MLLT3* group than other subtypes (41% vs. 16%, *p* = 0.004) [25]. A study by Issa et al. reported a more favorable prognosis for the *KMT2A*::*MLLT3* subgroup, with 5-year OS of 28%, although this difference did not reach statistical significance [26].

Early investigations have revealed that allo-HSCT in CR1 conferred a significant survival advantage for *KMT2A*-rearranged AML patients [28,32,33], and a recent study by Zhang et al. reported a notable survival advantage for *KMT2A*-rearranged AML patients who underwent HSCT, with OS of 77.3% compared to 31.7% in those receiving conventional chemotherapy (*p* < 0.001) [31]. However, Tamai et al. previously indicated that allo-HSCT in CR1 did not confer a substantial advantage in terms of disease-free survival (DFS) or OS for *KMT2A*-rearranged AML patients younger than 60 years [29]. These findings collectively highlight the need for further data on HSCT outcomes in relation to *KMT2A*-rearranged AML patients.

We explored the effects of HSCT across various *KMT2A*-rearranged subtypes. Our findings suggest that while the magnitude of benefit from HSCT may differ among distinct *KMT2A*-rearranged subtypes, HSCT in CR1 should generally be considered for all *KMT2A*-rearranged AML patients, regardless of the specific KMT2A fusion type. Specifically, our results support allo-HSCT for *KMT2A*::*MLLT3* patients. This recommendation is also supported by a recent report from Bataller et al., which demonstrated a significantly improved 2-year overall survival (OS) of 67% for *KMT2A*::*MLLT3* patients who underwent HSCT in CR1 [34], aligning with our observed survival outcomes. Additionally, our data indicated that *KMT2A*-rearranged patients aged 20 years or younger did not experience a significant survival advantage from HSCT. This finding challenges the assumption of HSCT benefit in *KMT2A*-rearranged AML, suggesting reduced transplant demand in younger patients with these genetic alterations. Our data advocate precision treatment strategy integrating genetic and clinical characteristics within this genetically diverse disease.

Since our study spanned 14 years, we examined the year of diagnosis to rule out any historical bias. We observed a significant improvement in OS for patients treated in the recent era (2017–2024) compared to the earlier era (2010–2016). This improvement likely reflects advances in supportive care and transplant techniques. To rule out potential bias, we included the year of diagnosis in our univariate and multivariate analyses. Although the year of diagnosis was significant for OS in the univariate analysis, this significance disappeared in the multivariate model for both OS and EFS. Additionally, in our *KMT2A*-rearranged cohort, there was no significant difference in the distribution of patients receiving different induction regimens across the various fusion partner subgroups. Therefore, we consider that the specific induction regimen did not significantly influence the survival outcomes in our survival analyses. This confirms that the survival differences among *KMT2A* subtypes are driven by the specific fusion partners, rather than by historical changes in treatment.

Furthermore, when comparing our results with cited historical cohorts, such as the research of CALGB and Grimwade et al. [12,13], several variations warrant consideration. Firstly, regarding analytical methods and sample size, our study utilized standard statistical techniques such as Kaplan–Meier and cox regression consistent with the cited literature [12,13,24,25,26,27,28,29,30,31]. Compared to referenced studies where *KMT2A*-rearranged cohorts typically ranged from 47 to 180 patients [12,13,24,25,26], our cohort (*n* = 181) represents a relatively large sample size with sufficient statistical power to yield robust prognostic insights. Secondly, regarding treatment heterogeneity, our cohort incorporated HHT-based induction (HAD regimen), which differs from the standard ‘7 + 3’ regimens typically used in Western cohorts [24,25,26,27]. While our internal analysis confirmed no significant survival difference between HAD and DA regimens in our cohort, these protocol variations preclude a direct head-to-head comparison of absolute survival rates. Thirdly, regarding follow-up duration, while some referenced studies reported 5-year outcomes, our study focused on 3-year OS and EFS. Given that *KMT2A*-rearranged AML is characterized by aggressive disease kinetics and early relapse [26,28,29], we consider our 3-year metrics sufficiently mature to capture the critical prognostic events and reflect the long-term survival trends. Despite these differences in sample sizes, therapeutic baselines, and analytical timepoints, the prognostic hierarchy of fusion partners observed in our cohort aligns with major international reports [30,31], underscoring that intrinsic biological characteristics are the principal driver of clinical outcomes.

Several limitations of our study should be acknowledged. Firstly, specific fusion partners could not be identified in 29.8% of patients, and NGS data were available for 70.7% (128/181) of the cohort due to the retrospective nature of the study. Secondly, a substantial proportion of patients could not be assigned to specific *KMT2A* rearrangement subtypes due to the limitations of FISH testing, potentially limiting the ability to detect subtype-specific differences. Thirdly, the relatively small sample sizes within certain subtype groups may have restricted the statistical power to detect subtle differences in survival outcomes. Fourthly, specific data on co-morbidities were not available for all patients; however, all included patients were deemed fit for intensive chemotherapy, suggesting a generally adequate performance status.

## 5. Conclusions

Our study provides additional evidence of the clinical heterogeneity of *KMT2A*-rearranged AML and highlights the critical role of subtyping in risk stratification and therapeutic decision-making. HSCT in CR1 significantly improved outcomes across different *KMT2A* fusion subtypes; however, the magnitude of benefit varied across patient age and subtypes. This clinical cohort study delineates treatment efficacy and survival outcomes in *KMT2A*-rearranged AML patients receiving intensive chemotherapy and HSCT, providing an evidence base for optimizing therapeutic strategies. Recent studies have identified menin inhibitors as a promising novel therapeutic approach for *KMT2A*-rearranged leukemia, offering a new option for patients with suboptimal responses to conventional therapies [35,36]. Future research should focus on elucidating the specific molecular mechanisms underlying the observed subtype-specific differences in survival, as well as exploring novel therapeutic strategies to improve outcomes for patients with *KMT2A*-rearranged AML.

## Figures and Tables

**Table 1:** Baseline characteristics of patients with *KMT2A*-rearranged AML.

Characteristics	All Subjects (*N* = 181)	HSCT in CR1 = NO (*N* = 107)	HSCT in CR1 = YES (*N* = 74)	*p* Value
Sex (n, %)				0.97
Male	89 (49.17)	52 (48.60)	37 (50.00)	
Female	92 (50.83)	55 (51.40)	37 (50.00)	
Age at diagnosis (median, range)	33 (13–65)	34 (15–65)	32.5 (13–55)	0.21
Median WBC, ×10^9^/L (median, range)	12.41 (0.54–361.64)	12.66 (0.54–361.64)	10.06 (0.73–336.23)	0.49
Partner (n, %)				0.11
ELL	24 (13.26)	9 (8.41)	15 (20.27)	
AFDN	27 (14.92)	20 (18.69)	7 (9.46)	
MLLT3	39 (21.54)	21 (19.63)	18 (24.32)	
MLLT10	25 (13.81)	14 (13.08)	11 (14.86)	
other	12 (6.63)	7 (6.54)	5 (6.76)	
unknown	54(29.83)	36 (33.64)	18 (24.32)	
Mutation (n, %)				
KRAS	41 (32.03)	25 (33.78)	16 (29.63)	0.75
NRAS	40 (31.25)	21 (28.38)	19 (35.19)	0.53
PTPN11	20 (15.62)	12 (16.22)	8 (14.81)	1.00
FLT3	19 (14.84)	14 (18.92)	5 (9.26)	0.21
WT1	10 (7.81)	5 (6.76)	5 (9.26)	0.74
ASXL1	7 (5.47)	3 (4.05)	4 (7.41)	0.45
CCND3	5 (3.91)	3 (4.05)	2 (3.70)	1.00
SETD2	5 (3.91)	5 (6.76)	0 (0.00)	0.07
U2AF1	4 (3.13)	3 (4.05)	1(1.85)	0.64

Abbreviation: WBC, white blood cell; HSCT, hematopoietic stem cell transplantation.

## Data Availability

The datasets analyzed for this study are available from the corresponding author upon reasonable request.

## Supplementary Materials

The following supporting information can be downloaded at: https://www.mdpi.com/article/10.3390/cancers18030401/s1, Figure S1: Distribution of KMT2A fusion partners in this study cohort (*n* = 181); Figure S2: Kaplan–Meier curves of OS and EFS between patients with specific KMT2A rearranged subtypes and the remaining patients; Figure S3: Baseline survival outcomes of the entire cohort; Figure S4: Survival analysis stratified by fusion partners after setting HSCT as a censoring event; Figure S5: Kaplan–Meier survival analysis stratified by the year of diagnosis; Table S1: List of 51 leukemia-associated fusion genes covered by the multiplex RT-PCR screening panel; Table S2: Genes included in the targeted next-generation sequencing panel; Table S3: Baseline characteristics stratified by KMT2A-rearranged fusion partners; Table S4: Univariate cox regression analysis of factors associated with OS and EFS in KMT2A-rearranged AML Patients; Table S5: Multivariate cox regression analysis of factors associated with OS and EFS in KMT2A-rearranged AML Patients.

## Abbreviations

The following abbreviations are used in this manuscript:

AML	Acute Myeloid Leukemia
allo-HSCT	allogeneic hematopoietic stem cell transplantation
CR	Complete Remission
CR1	First complete remission
CI	Confidence Interval
DFS	Disease-free survival
EFS	Event-free survival
ELN	European Leukemia Net
FISH	Fluorescence In Situ Hybridization
HR	Hazard ratio
IGV	Integrative Genomics Viewer
KMT2A	Lysine methyltransferase 2A
MLL	Mixed lineage leukemia
NCCN	National Comprehensive Cancer Network
NGS	Next-generation sequencing
NR	Not reached
OS	Overall survival
RT-PCR	Reverse Transcription Polymerase Chain Reaction
WBC	White blood cell
