# Supplementary material for: Impact of Fusion Partners and Transplantation Benefit in Intensively Treated KMT2A-Rearranged Acute Myeloid Leukemia
Source: Cancers (Basel). 2026 Jan 27;18(3):401. doi: 10.3390/cancers18030401 (PMC12896996; doi:10.3390/cancers18030401)
Supplement: Supplementary file 1 [file cancers-18-00401-s001.zip › cancers-4114450-SM/cancers-4114450-figure/spfigure and figure legend.pdf]

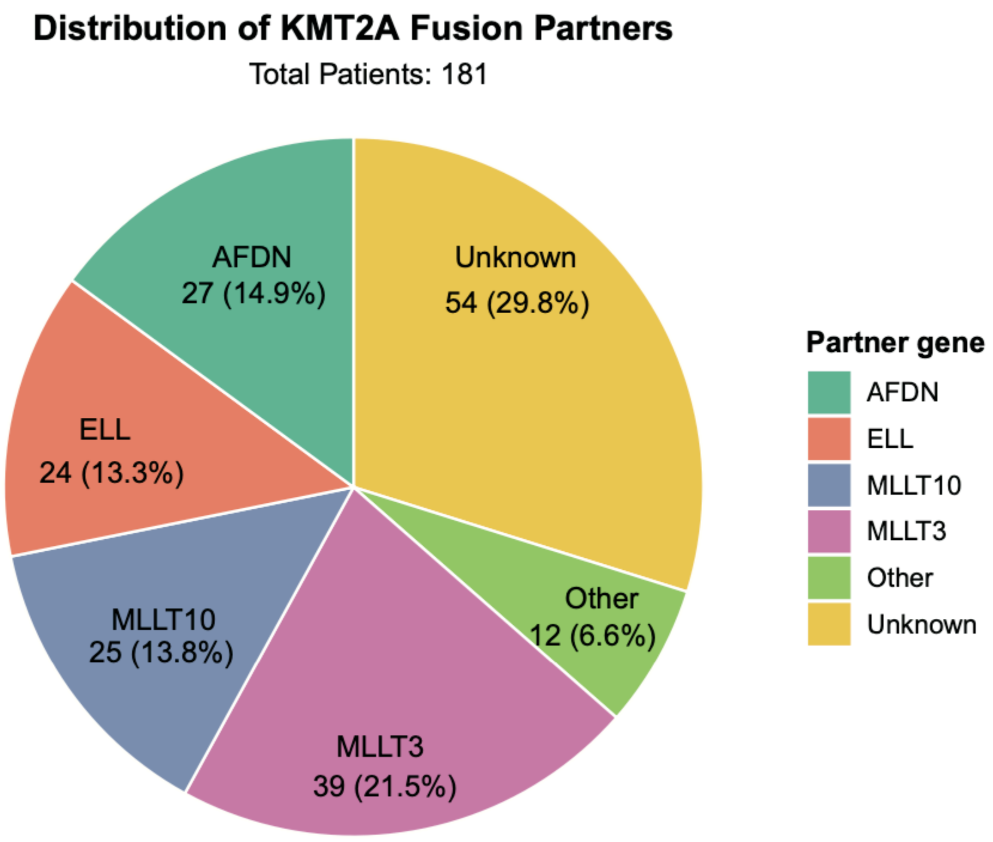

Supplementary figure S1. Distribution of *KMT2A* fusion partners in this study cohort (n=181). The chart displays the proportional distribution of different *KMT2A* fusion partners across the cohort. *MLLT3* (21.5%) was the most common identified partner gene, followed by *AFDN* (14.9%), *MLLT10* (13.8%) and *ELL* (13.3%).

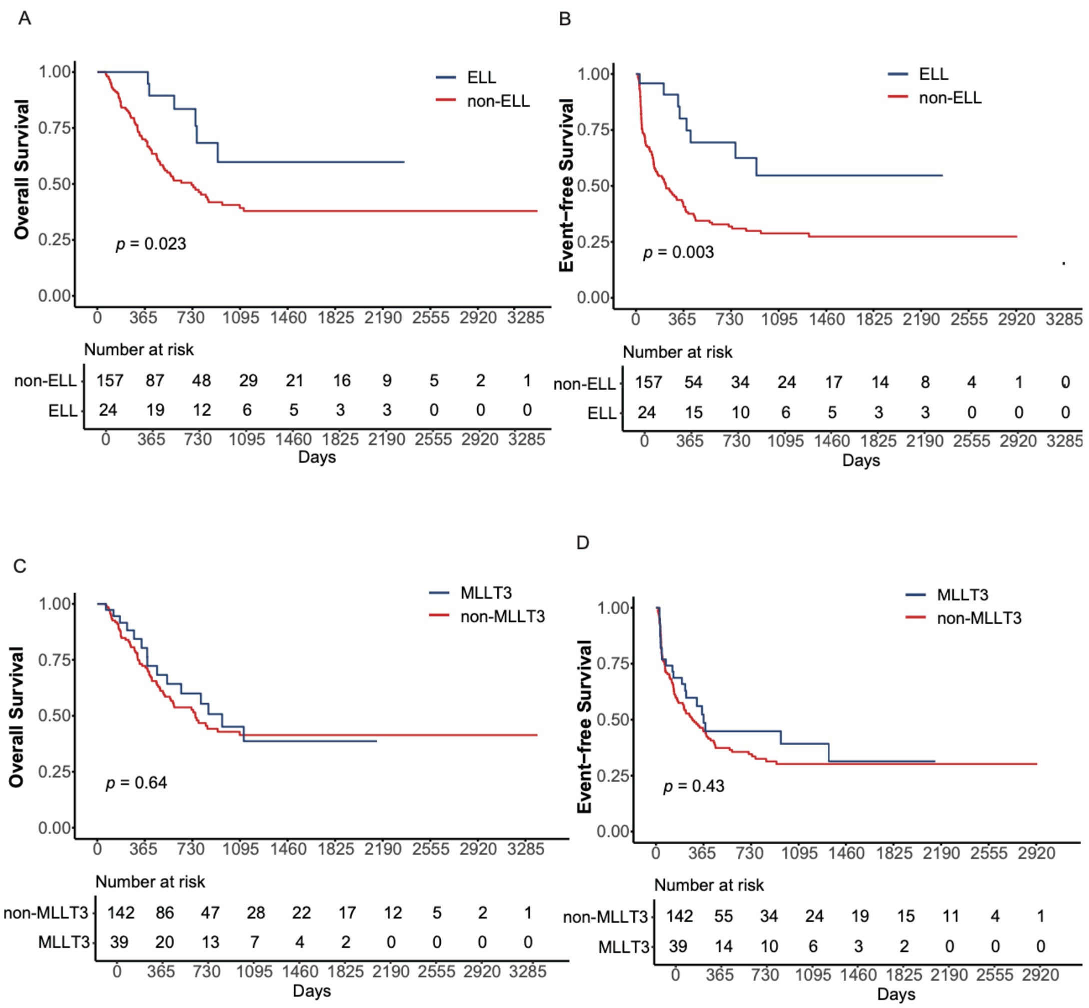

Supplementary figure S2. Kaplan-Meier curves of OS and EFS between patients with specific *KMT2A* rearranged subtypes and the remaining patients. (**A, B**) Comparison of OS (**A**) and EFS (**B**) between patients with and without *KMT2A*::*ELL*. (**C, D**) Comparison of OS (**C**) and EFS (**D**) between patients with and without *KMT2A*::*MLLT3*.

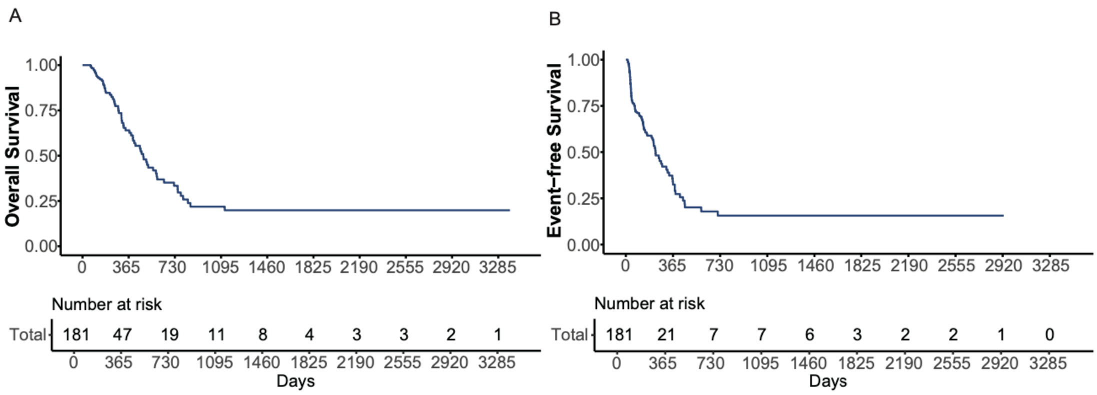

Supplementary figure S3. Baseline survival outcomes of the entire cohort.

Kaplan-Meier curves showing the OS (**A**) and EFS (**B**) for the entire cohort.

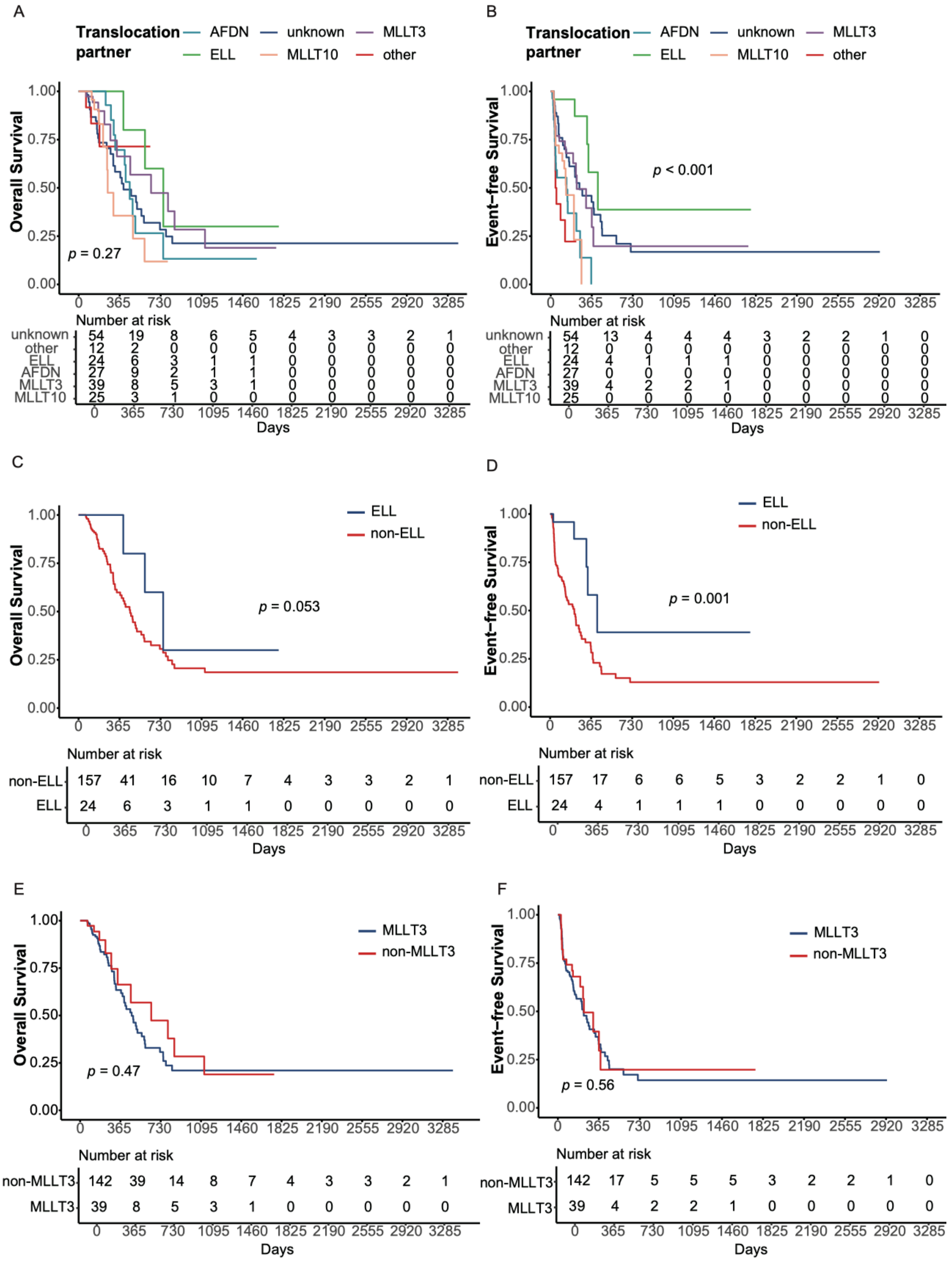

Supplementary figure S4. Survival analysis stratified by fusion partners after setting transplantation as a censoring event. (**A, B**) Kaplan-Meier curves of OS (**A**) and EFS (**B**) stratified by fusion partner subgroups. (**C, D**) Comparison of OS (**C**) and EFS (**D**) between patients with and without *KMT2A*::*ELL*. (**E, F**) Comparison of OS (**E**) and EFS (**F**) between patients with and without *KMT2A*::*MLLT3*. The p-values indicate the global significance across all subgroups, calculated using the Log-rank test.

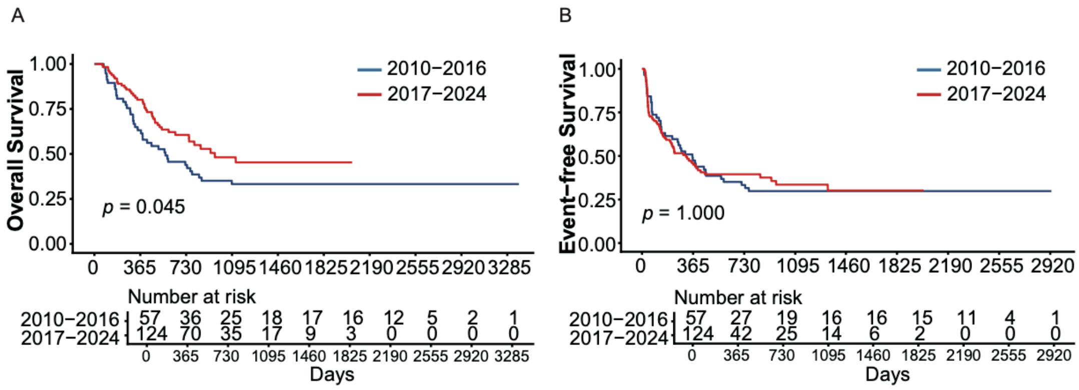

Supplementary figure S5. Kaplan-Meier survival analysis stratified by the year of diagnosis. Comparison of (**A**) OS and (**B**) EFS between patients diagnosed in the earlier era (2010–2016) and the recent era (2017–2024).
