# Supplementary figures and images for: Impact of Fusion Partners and Transplantation Benefit in Intensively Treated KMT2A-Rearranged Acute Myeloid Leukemia
Source: Cancers (Basel). 2026 Jan 27;18(3):401. doi: 10.3390/cancers18030401 (PMC12896996; doi:10.3390/cancers18030401)

## Supplementary Figure 1.

### Distribution of KMT2A Fusion Partners

Total Patients: 181

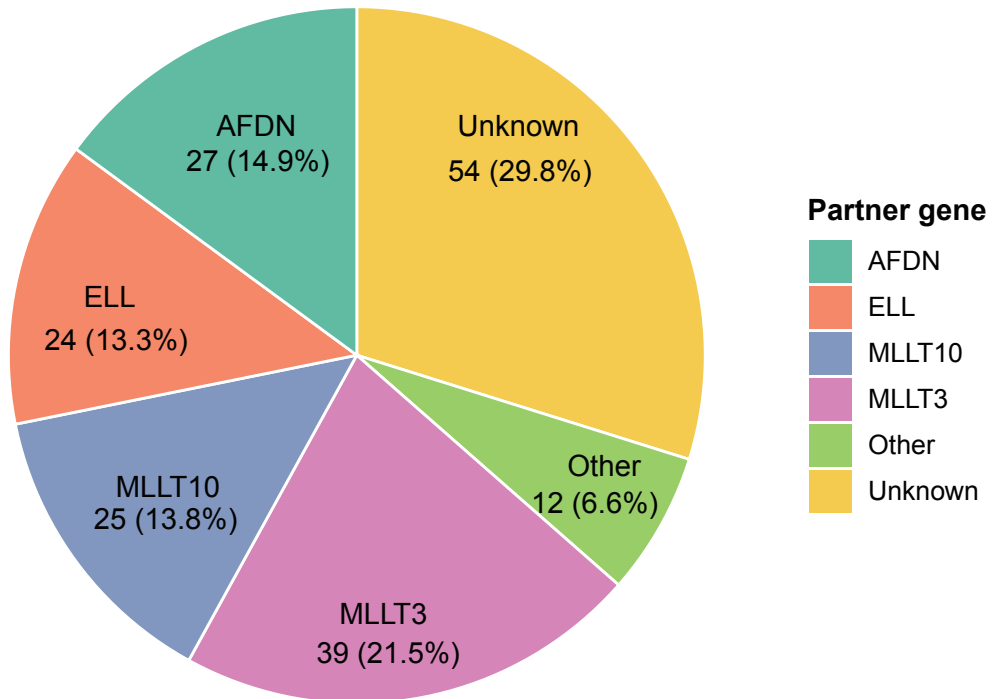

Supplement: Supplementary file 1 [file cancers-18-00401-s001.zip › cancers-4114450-SM/cancers-4114450-figure/figure S1.pdf]

Supplementary figure 1

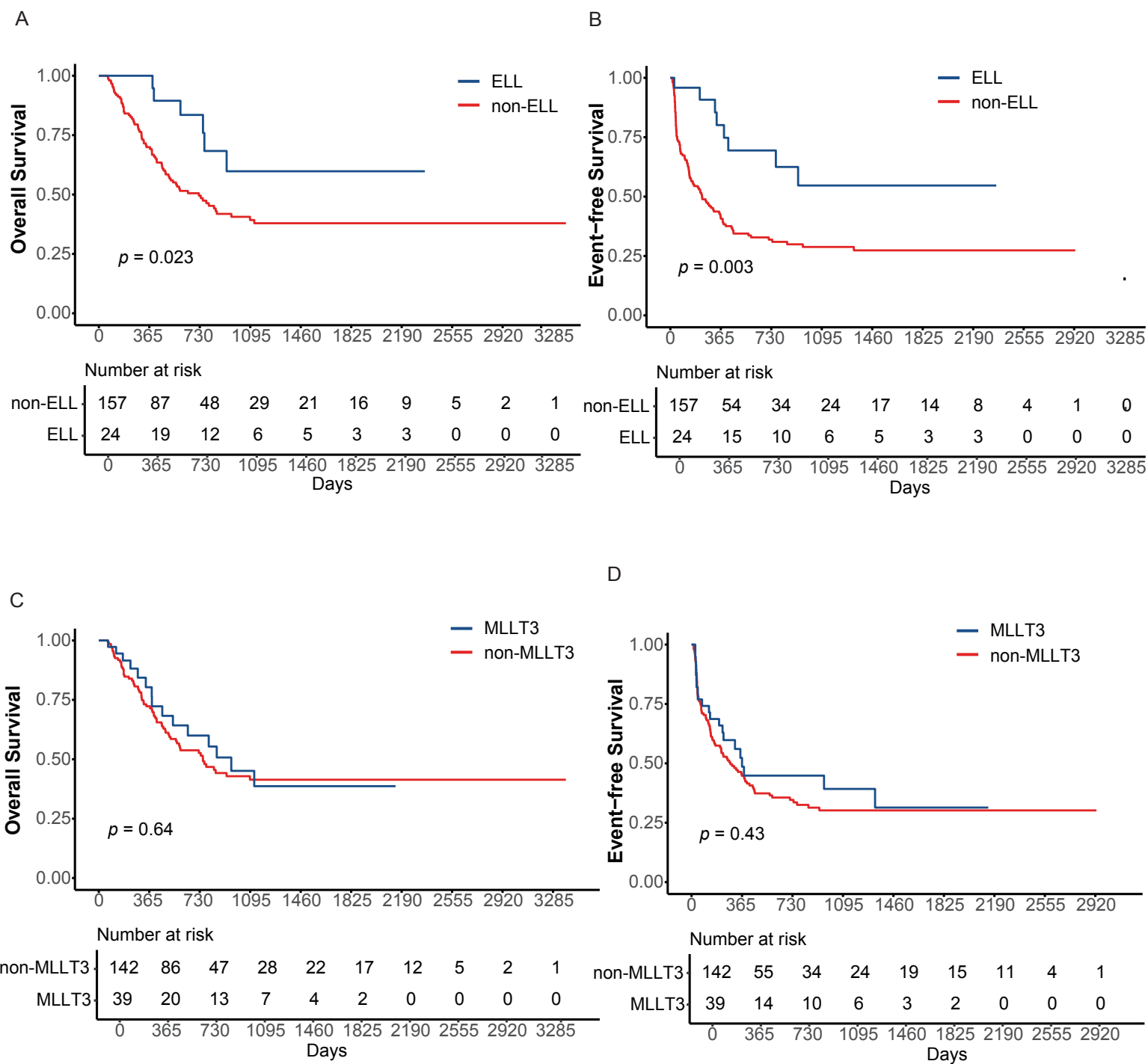

Supplement: Supplementary file 1 [file cancers-18-00401-s001.zip › cancers-4114450-SM/cancers-4114450-figure/figure S2.pdf]

Supplementary figure 2

A

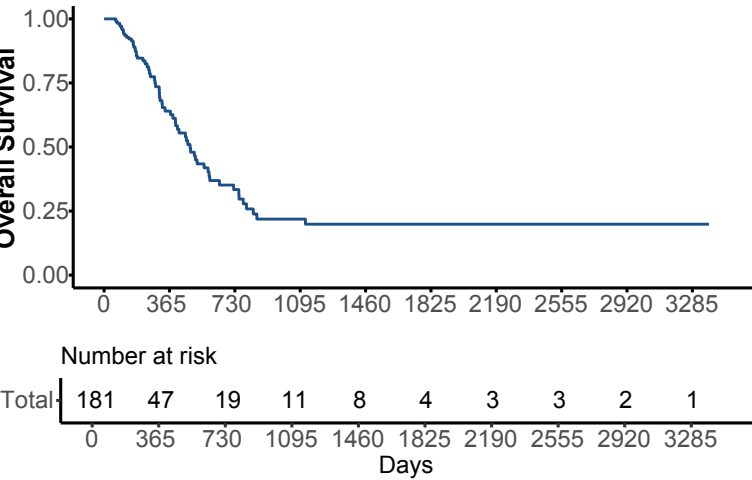

B

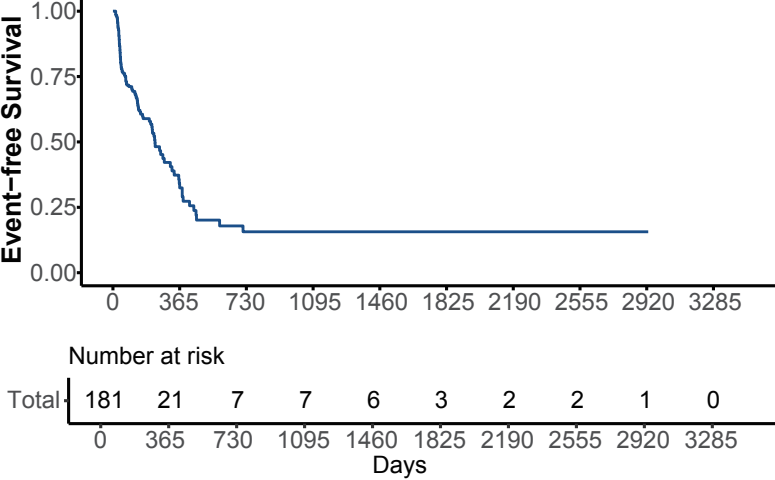

Supplement: Supplementary file 1 [file cancers-18-00401-s001.zip › cancers-4114450-SM/cancers-4114450-figure/figure S3.pdf]

Supplementary figure 3

A

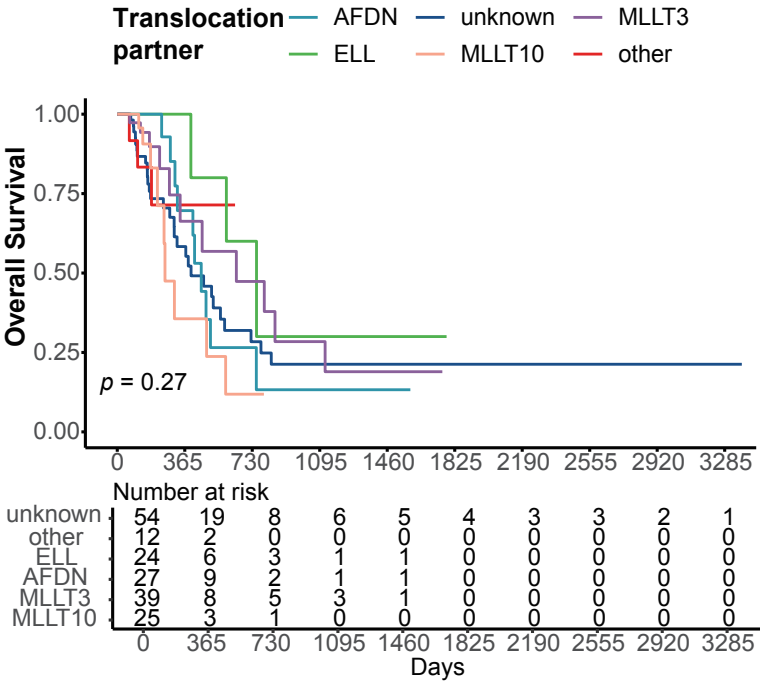

B

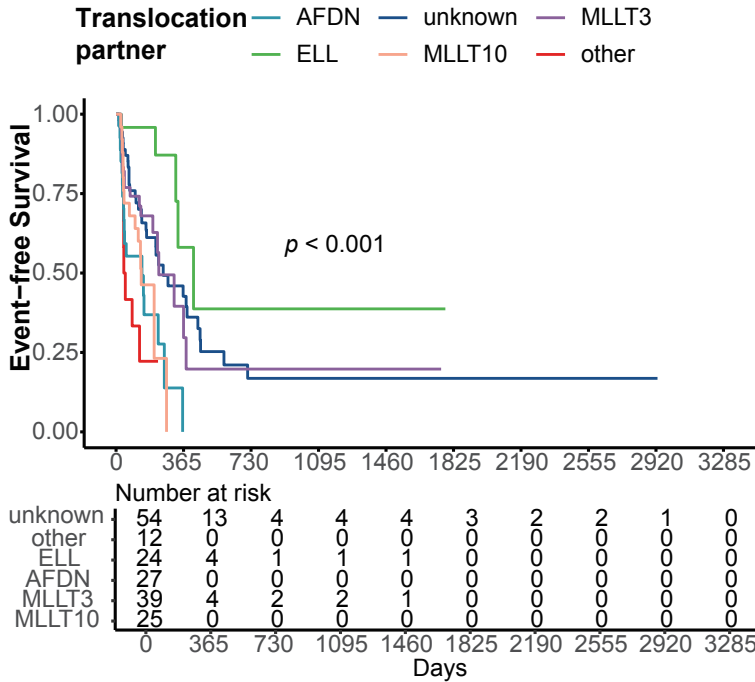

C

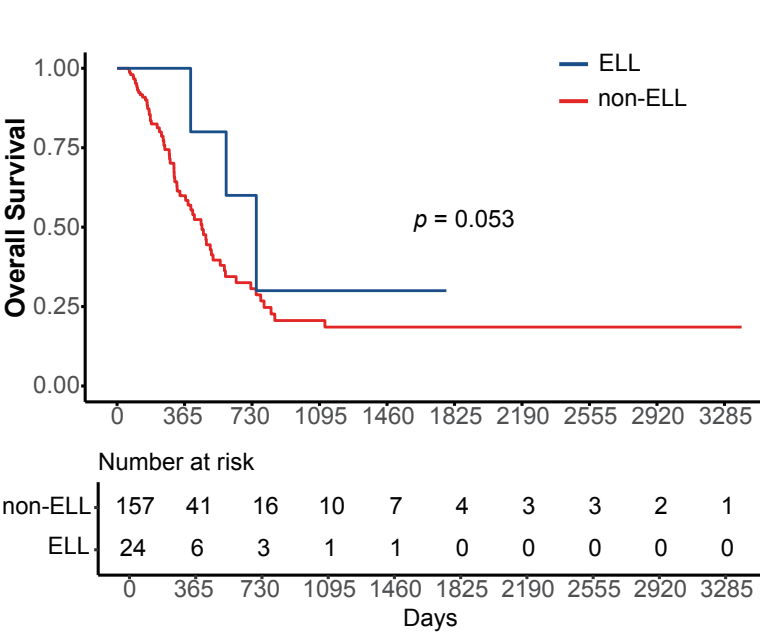

D

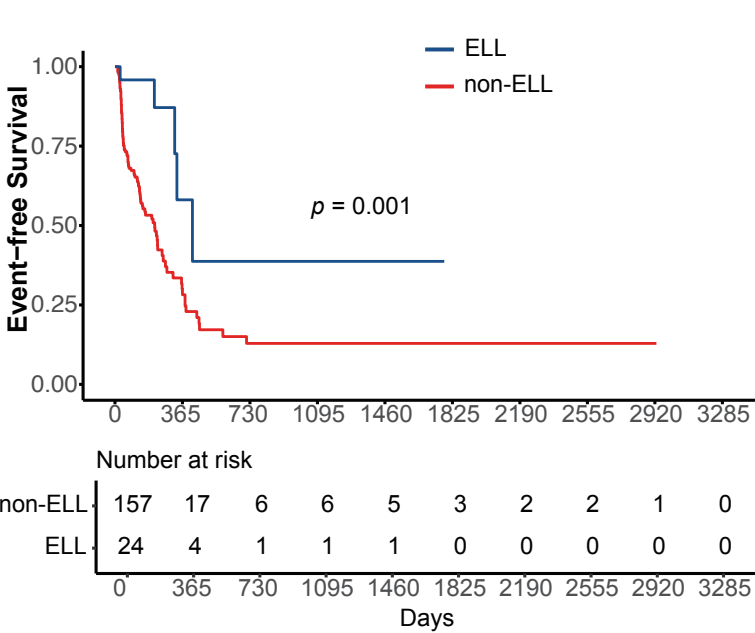

E

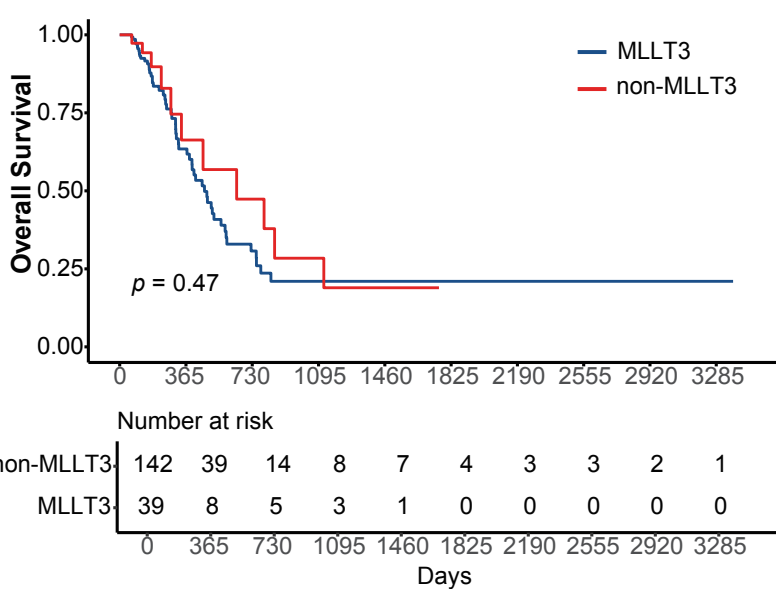

F

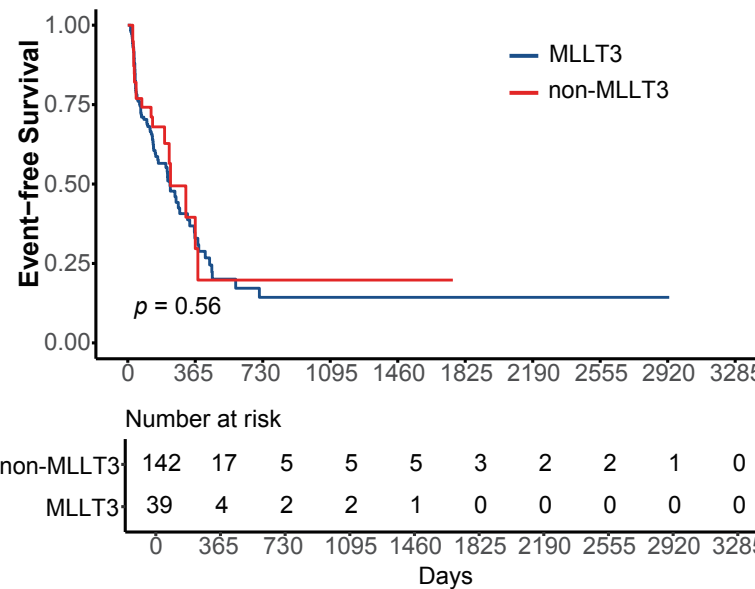

Supplement: Supplementary file 1 [file cancers-18-00401-s001.zip › cancers-4114450-SM/cancers-4114450-figure/figure S4.pdf]

Supplementary Figure S5

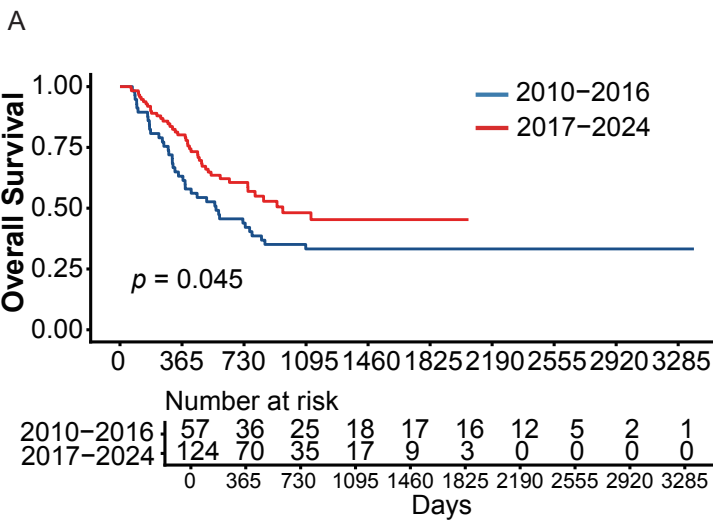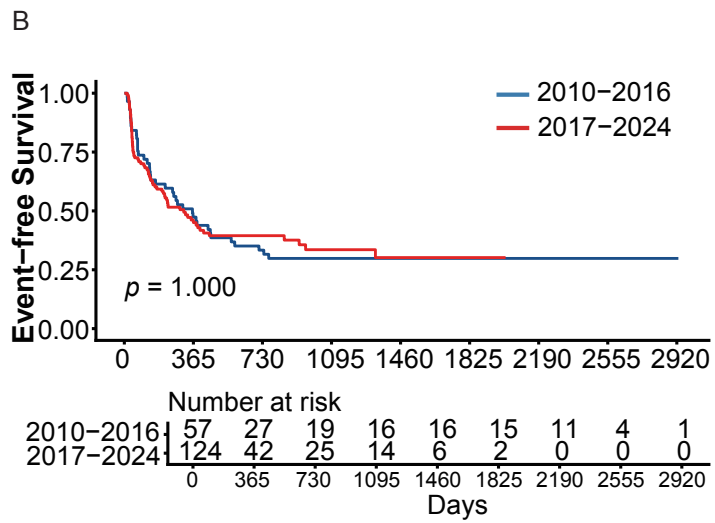

Supplement: Supplementary file 1 [file cancers-18-00401-s001.zip › cancers-4114450-SM/cancers-4114450-figure/figure S5.pdf]
